# Adult-Onset T-Cell Acute Lymphoblastic Lymphoma-Leukemia Presenting With Petechial Rash: A Case Report

**DOI:** 10.7759/cureus.67744

**Published:** 2024-08-25

**Authors:** Kelly Kimball, Vissy Elad, Edward J Hamad, Christopher Wasco

**Affiliations:** 1 Internal Medicine, OhioHealth Riverside Methodist Hospital, Columbus, USA; 2 College of Medicine, Northeast Ohio Medical University, Rootstown, USA

**Keywords:** oncology, dermatology, chemotherapy, petechial rash, t-cell lymphoma

## Abstract

T-cell acute lymphoblastic lymphoma-leukemia (T-ALL) is a rare neoplastic disease with presenting symptoms that are often non-specific. As such, accurate diagnosis requires high clinical suspicion and assessment of bone marrow aspirate with flow cytometry and morphology. While remission is achievable in most patients, the five-year survival rate is only 48% despite treatment. The standard chemotherapy regimen is referred to as hyper-CVAD, where CVAD stands for cyclophosphamide, vincristine sulfate, Adriamycin (doxorubicin), methotrexate, and dexamethasone. In this study, we describe a 38-year-old female who presented with atraumatic abdominal bruising and a petechial rash on the upper and lower extremities. Imaging revealed a 16 cm anterior mediastinal mass and a bone marrow and mediastinal mass biopsy confirmed a diagnosis of T-ALL. The patient received hyper-CVAD cycle 1A with several complications but ultimately achieved remission after four cycles. Here, we stress the importance of broadening differentials for new-onset petechial rashes in adults to include systematic lymphomas, such as T-ALL, and the need for early recognition so patients can receive timely treatment.

## Introduction

T-cell acute lymphoblastic lymphoma-leukemia (T-ALL) is a rare neoplastic disease with an incidence of 4,000 cases in the United States each year [1]. The majority of cases appear in childhood, between the ages of two and 10 years. Within the adult population, this cancer is even rarer, with T-ALL accounting for approximately 25% of all cases of ALL in adults [2]. A retrospective population-based analysis of the Surveillance, Epidemiology, and End Results Program (SEER) database between 2001 and 2014 showed the incidence of T-ALL in the United States adult population to be 0.13 cases per 100,000 [3]. Initial presentations of T-ALL are non-specific and include symptoms, such as weight loss, weakness, lymphadenopathy, easy bruising, and skin pallor [1]. Diagnosis involves the assessment of bone marrow aspirate and its subsequent flow cytometry, morphology, and above all, adequate clinical suspicion. The main immunophenotype markers in T-ALL include terminal deoxynucleotidyl transferase (TdT) and CD3, with other markers, such as CD1a, CD2, CD4, CD5, CD7, and CD8, being variably expressed [4]. To achieve remission of T-ALL, patients are typically treated with multiagent intrathecal and intravenous chemotherapy delivered in intensive courses over two to three years with a possibility for adjunct post-remission radiation therapy in cases with CNS involvement [5]. One prospective trial of 334 patients found that 94% of T-ALL patients achieved complete remission after 2-phase induction over eight weeks and showed a five-year overall survival rate of 48%; patients were followed for 10 years [6,7]. Many patients with T-ALL receive treatment with a regimen commonly referred to as hyper-CVAD, where CVAD stands for cyclophosphamide, vincristine sulfate, Adriamycin (doxorubicin), methotrexate, and dexamethasone [4].

In this report, we describe a 38-year-old female who presented with atraumatic abdominal bruising and a petechial rash on the upper and lower extremities. She was found to have T-ALL and a 16 cm anterior mediastinal mass. Herein, we stress the importance of broadening differentials for new-onset petechial rashes in otherwise healthy adults to include systemic diseases, such as hematologic malignancies.

## Case presentation

**Figure 1:**
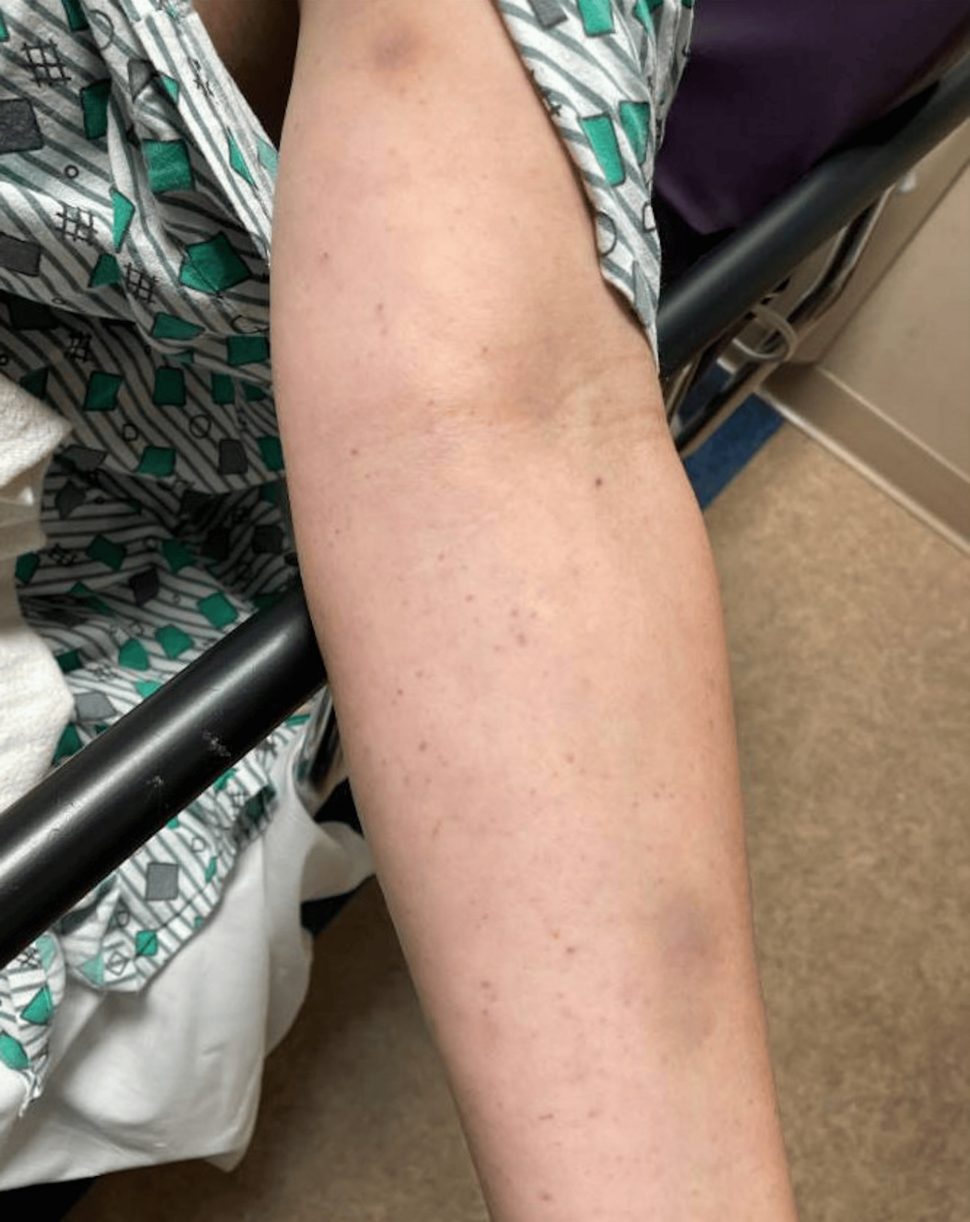
Multiple, atraumatic, non-blanching petechiae involving the upper extremities.

**Figure 2:**
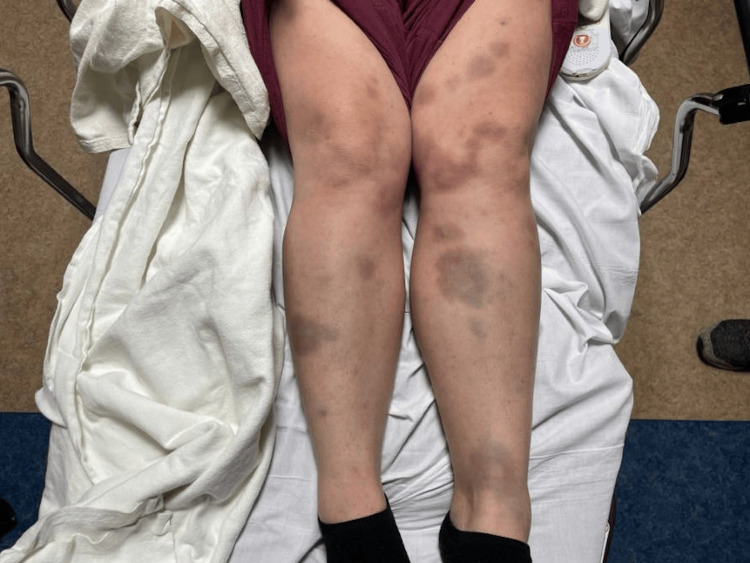
Generalized petechial eruption with non-palpable purpura and ecchymoses involving the bilateral lower extremities.

A 38-year-old female with no significant medical history presented with a six-day history of progressive atraumatic abdominal bruising and petechial rash on her upper and lower extremities in October 2023 (Figures 1, 2). These symptoms were accompanied by drenching night sweats, low-grade fevers, chest heaviness, and heavy menstruation.

**Figure 3:**
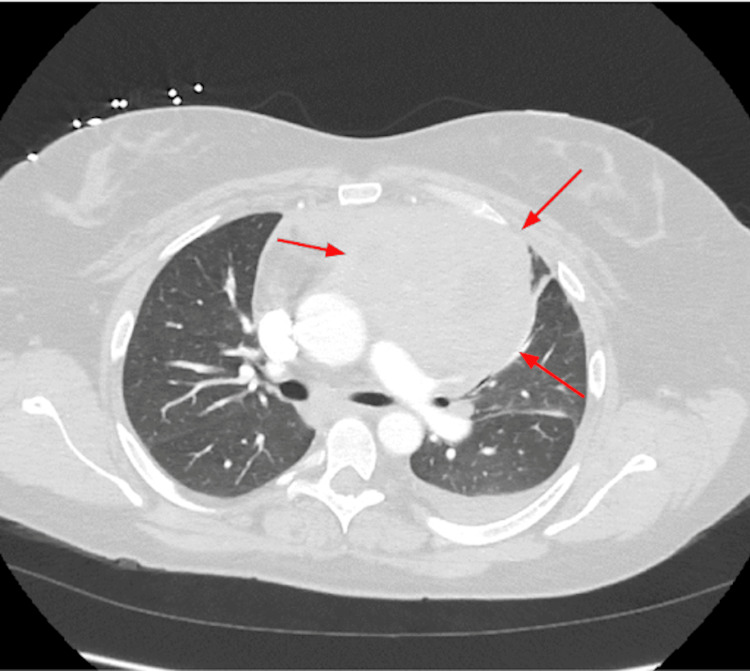
A homogeneous anterior mediastinal mass with local mass effect measuring 16 cm (arrows).

Laboratory studies showed a white blood cell count of 42,000/μL and a platelet count of 8,000/μL (Table 1). A computed tomography (CT) pulmonary embolism study was negative for pulmonary embolism but remarkable for a large soft tissue mass in the anterior mediastinum measuring up to 16 cm (Figure 3).

**Table 1:** Abnormal patient laboratory findings at presentation.

Test	Patient values	Reference range
White blood cells (count/μL)	42,000	4,500-11,000
Platelet count (count/μL)	8,000	150,000-450,000
Lactate dehydrogenase (U/L)	>2,500	140-280
Serum uric acid (mg/dL)	13.4	2.7-7.3
Serum fibrinogen (mg/dL)	493	200-400

**Figure 4:**
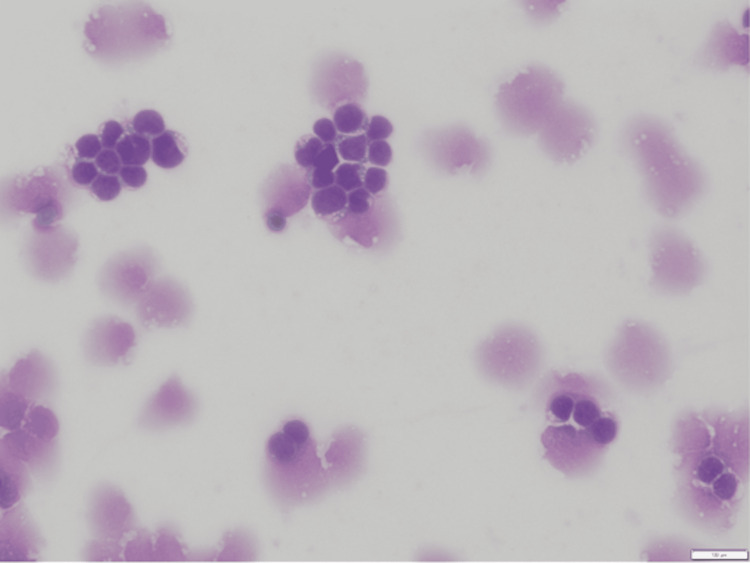
Peripheral blood smear with increased circulating lymphoblasts (10×).

Core needle biopsy of the mediastinal mass demonstrated diffuse effacement of the biopsy by immature mononuclear cells, compatible with lymphoblasts. By immunohistochemistry analysis, the neoplastic cells had strong nuclear staining with TdT, were positive for CD99, weakly positive for CD5, and positive for CD3 in a pattern consistent with T-ALL. Initial bone marrow biopsy of the iliac crest showed effacement with lymphoblasts positive for strong nuclear TdT and cytoplasmic CD3 as well as CD99 and CD1a, consistent with T-ALL. There was an absence of significant keratin staining with a keratin AE1/AE3 immunostain, which is again consistent with T-ALL. A potential diagnosis of acute T-cell leukemia/lymphoma (ATLL) was considered due to the increased number of circulating lymphoblasts in the peripheral blood smear. However, this diagnosis was ruled out based on negative testing for human T-lymphotropic virus types 1 and 2 (HTLV-1/2) infection (Figure 4). She ultimately received the diagnosis of adult-onset T-ALL given her bone marrow biopsy results. The patient received hyper-CVAD cycle 1A which includes cyclophosphamide, vincristine, doxorubicin, and dexamethasone. Her hospitalization was complicated by subdural hemorrhage, acute kidney injury, vaginal bleeding, and mucositis. She was discharged home in stable condition 19 days later; however, she did have some lingering sinus congestion and voice hoarseness.

The patient presented to the hospital two days after discharge with complaints of gland swelling, epistaxis, ear soreness, and ongoing sinus congestion and eventually tested positive for parainfluenza 2. She was admitted and treated with intravenous (IV) ampicillin-sulbactam, and then later treated with IV vancomycin. She went on to receive cycle 1B of hyper-CVAD five days later, which included methotrexate and cytarabine and was tolerated well. She received intrathecal (IT) chemotherapy on day two with methotrexate as prophylaxis for leptomeningeal spread. She was discharged home receiving day seven (D7) IT chemotherapy with cytarabine three days later as an outpatient. Cerebrospinal fluid was negative for malignant cells. She recovered well with symptomatic management and did not require readmission. After four cycles of hyper-CVAD, a bone marrow biopsy of the iliac crest was obtained for restaging of her disease; however, the aspirate did not show any T-lymphoblasts identified via flow cytometry, supporting the attainment of molecular remission of her T-ALL. Positron emission tomography (PET)-CT restaging 1.5 months later showed almost complete resolution of her mediastinal mass; therefore, consolidative radiation was not pursued. The patient remains in remission and is receiving two years of prednisone, vincristine, methotrexate, and mercaptopurine (POMP) maintenance therapy with pegylated (PEG) asparaginase intensification.

## Discussion

Here, we present an innocuous case of T-ALL with a constellation of non-specific findings. We stress the importance of broadening differentials for new-onset petechial rashes in adults to include systematic lymphomas, such as T-ALL, and highlight the need for early recognition so patients can receive timely treatment. The patient received hyper-CVAD cycle 1A and had several complications, but ultimately achieved remission after four cycles. This case is a strong example of how high clinical suspicion for relatively rare etiologies of disease can reduce patient morbidity and mortality. The median overall survival of T-ALL in adult populations is estimated to be 34 months; one of the potential contributing factors to this statistic is delay/interruption of therapy, such as early relapse, treatment-related toxicity, and non-compliance [3]. In cases such as these, subtle dermatologic findings like this bear the potential to play a major role in elucidating the final diagnosis [7,8]. While the lymph nodes and mediastinum are sites most frequently implicated in T-ALL, other sites like the skin or kidneys should not be subject to a lower threshold of diagnostic scrutiny due to their lower frequency of involvement in this genre of neoplastic processes [9]. It is estimated that between 5% and 8% of all visits to the emergency department are due to dermatologic concerns, and while petechiae and purpura can often be non-specific skin findings, they should not be overlooked by providers [10]. In patients with T-ALL who develop petechial rashes, the legs are the most commonly involved location for lesions to develop, which aligns with this patient’s presentation [11]. Interestingly, one study examined over 5,000 patients with ALL and found that spleen and liver enlargement are prevalent in T-ALL, findings that were not observed in the present case [12]. This may be attributed to the patient’s age, as these findings become less frequent in older populations, possibly due to age-related atrophy of lymphoid organs [12]. There have been other instances where a patient’s presenting symptoms do not immediately appear to be the result of a hematologic malignancy. For example, the case of a 19-year-old male with T-cell lymphoblastic lymphoma who presented with acute renal failure, lactic acidosis, and a very near normal complete blood count, highlighting the diverse presentations of T-ALL and the risk for misdiagnosis [13]. Cases like these serve as reminders to consider more than the most probable diagnosis and reinforce the value of thorough investigation. In an otherwise healthy person, a chief complaint of petechial/purpuric rash warrants further workup to diagnose potential systemic diseases, such as hematologic malignancies. Fortunately for this patient, she was able to achieve molecular remission and tumor regression, likely due to early identification and treatment.

## Conclusions

This study highlights several key take-home points, with one of the most notable being the importance of having a broad differential for adult-onset petechial rashes. These differentials may include T-ALL, which often presents with non-specific findings and needs a high clinical suspicion for diagnosis. In otherwise healthy individuals, the onset of a new petechial/purpuric rash provides a rationale for further investigation to diagnose potential systemic diseases, including hematologic malignancies. The sooner a systemic work-up is started by the healthcare team, the greater the likelihood of successful clinical outcomes, as was experienced in the present case.
